# Regret among primary care physicians: a survey of diagnostic decisions

**DOI:** 10.1186/s12875-020-01125-w

**Published:** 2020-03-17

**Authors:** Beate S. Müller, Norbert Donner-Banzhoff, Martin Beyer, Jörg Haasenritter, Angelina Müller, Carola Seifart

**Affiliations:** 1 Institute of General Practice, Goethe University Frankfurt, Theodor-Stern-Kai 7, 60590 Frankfurt am Main, Germany; 2 Department of General Practice / Family Medicine, University of Marburg, Karl-von-Frisch-Strasse 4, 35043 Marburg, Germany; 3 Department of Pneumology, and Ethics Commission, University of Marburg, Baldingerstrasse, 35032 Marburg, Germany

**Keywords:** Regret, Diagnostic error, Uncertainty, Primary health care, General practice, Patient safety

## Abstract

**Background:**

Experienced and anticipated regret influence physicians’ decision-making. In medicine, diagnostic decisions and diagnostic errors can have a severe impact on both patients and physicians. Little empirical research exists on regret experienced by physicians when they make diagnostic decisions in primary care that later prove inappropriate or incorrect. The aim of this study was to explore the experience of regret following diagnostic decisions in primary care.

**Methods:**

In this qualitative study, we used an online questionnaire on a sample of German primary care physicians. We asked participants to report on cases in which the final diagnosis differed from their original opinion, and in which treatment was at the very least delayed, possibly resulting in harm to the patient. We asked about original and final diagnoses, illness trajectories, and the reactions of other physicians, patients and relatives. We used thematic analysis to assess the data, supported by MAXQDA 11 and Microsoft Excel 2016.

**Results:**

29 GPs described one case each (14 female/15 male patients, aged 1.5–80 years, response rate < 1%). In 26 of 29 cases, the final diagnosis was more serious than the original diagnosis. In two cases, the diagnoses were equally serious, and in one case less serious. Clinical trajectories and the reactions of patients and relatives differed widely. Although only one third of cases involved preventable harm to patients, the vast majority (27 of 29) of physicians expressed deep feelings of regret.

**Conclusion:**

Even if harm to patients is unavoidable, regret following diagnostic decisions can be devastating for clinicians, making them ‘second victims’. Procedures and tools are needed to analyse cases involving undesirable diagnostic events, so that ‘true’ diagnostic errors, in which harm could have been prevented, can be distinguished from others. Further studies should also explore how physicians can be supported in dealing with such events in order to prevent them from practicing defensive medicine.

## Background

Regret is an emotion that occurs when a person realises or imagines that the current situation would be better if a decision he or she has made had been different. It can be defined as “negative emotions connected to cognitions about how past actions might have achieved better outcomes” [1]. Regret is often associated with self-blame and a strong wish to undo the current situation. It is primarily a backward-looking emotion that reflects the unfavourable evaluation of a decision [2]. Studies show that experienced as well as anticipated regret have an impact on human decision-making, as human beings anticipate future regret and try to apply strategies to avoid it [3]. Up to now, previous research has concentrated on preferential tasks, such as consumer decisions, [2] and much less work has invested in inferential tasks, of which a medical diagnosis is a typical example [4]. Formal conceptualisations of regret have been proposed for health care as well, [5–9] and a fair amount of studies has targeted patients’ regret after decisions affecting their health [10]. Little empirical research exists though on the extent to which physicians feel regret after making diagnostic decisions. This is surprising, since decisions made by health professionals can have grave consequences for patients and themselves, resulting in ample potential for regret [11, 12].

On the one hand, regret typically accompanies uncertainty. In a fully deterministic and knowable world, the outcome of decisions would always be known and there would be no reason for regret. In primary care, uncertainty is higher than in other medical disciplines [13–15]. Rare and serious diseases often present in early, atypical stages, but still have to be distinguished from other far more frequent benign and self-limiting conditions. On the other hand, regret also accompanies initial certainty and confidence. The consecutive feeling of disappointment and underachievement can compromise physicians’ personal image, thus, leading to repeated regret and encouraging the negative effects of regret to outweigh [1]. Since GPs (general practitioners) often act as gatekeepers and coordinate specialist treatment, their diagnostic decisions crucially influence the further diagnostic path and thereby the trajectory of the disease [16, 17].

We conducted a survey among general practitioners to explore the role that regret plays following diagnostic decisions.

## Methods

### Design

We conducted a qualitative study using an online questionnaire. We asked participants to describe cases when the final diagnosis had differed from what they had suspected during the first consultation. We were particularly interested in original and final diagnoses, possible harm to patients and the reactions of physicians, patients and families.

### Recruitment of participants

Between March and July 2016, we recruited a sample of primary care physicians. We distributed the link to the online questionnaire at meetings taking place in the academic departments of general practice at the Universities of Marburg and Frankfurt (150 participants), as well as via primary care mailing lists (500 recipients) and in the newsletter of a German critical incident reporting system for primary care (700 recipients) [18]. The link was also included in an article on diagnostic errors in a German journal published by the Union of Primary Care Physicians (circulation of 60,000 copies).

### Target cases

We specified that cases in which the final diagnosis differed from the original assumption were of interest. The case may also have resulted in an investigation by professional bodies, or litigation. In- and exclusion criteria are specified below:

Inclusion criteria:

- The participating physician was responsible for the original diagnosis, but a different explanation (diagnosis) was provided later.
- Treatment was delayed, which may have resulted in additional harm to the patient.

Exclusion criteria:

- The clinician regarded his/her diagnosis as suboptimal but as having no negative consequences for the patient (the latter defined as delayed treatment at the very least).
- The event occurred more than 5 years ago.

### Questionnaire and data collection

A preliminary questionnaire was developed for this study by NDB, JH and MB on the basis of their own previous work in the field [19]. To check clarity, comprehensiveness, relevance of response categories and the length of time required to fill in the questionnaire, it was tested by BM during four think-aloud interviews with primary care physicians. The revised version consisted of 17 questions and took 10 to 15 min to fill out (see Additional file 1). The questionnaire was presented using the online tool LimeSurvey™ [20].

On the first page, we advised participants to enter only anonymised data and no features that might make individual patients or health care professionals identifiable. We also provided information on data handling and confidentiality procedures. Further questions concerned details of the case, including the initial presentation, and the subsequent course of events. We wanted to know how physicians reacted and felt when it transpired that their diagnostic decisions may have been incorrect. We also encouraged them to elaborate on the reactions of patients, relatives and other physicians involved in the case (see Table 1). Finally, we asked for demographic details and information on the workplace. Physicians participated voluntarily and received no payment or other compensation.

**Table 1:** Questionnaire (excerpt)

Nr.	Questions about the case and subsequent reactions
1	Please describe your original impression of the patient. At this point, please only describe the situation during the first consultation, as you will be asked to describe the further course of events down below. Please consider: - Where it took place? Practice, hospital etc. - The patient’s age and gender - Previous diseases and other relevant factors - Current symptoms and findings - Your diagnostic assessment - Any other measures you may have initiated (further examination, referral, hospitalisation, therapy).
2	How long ago did the contact with the patient occur?
3	Further course of events: please describe what happened later.
4	Your reaction. What did you do then? How did you react to the situation emotionally?
5	Please describe the reaction of the patient and/or relatives. Please think of the following possibilities, which are not mutually exclusive: - Conversation with the patient and/or relatives - Patient changed doctor - Patient got in contact with a hearing officer/mediator - Civil or criminal proceedings

### Data analysis

The main objective of the analysis was to collect descriptions of the range of cases, as well as possible reactions and behaviours. In accordance with the framework of Barroso & Sandelowski, this was mostly a thematic survey [21]. In a first step, we displayed verbal accounts in paraphrased form for within- and cross-case analysis. When additional themes were identified, further fields were added. Two researchers analysed the data (CS, NDB). They discussed discrepancies in data display and related conclusions. The latter were also discussed in a group containing all the authors, and consensus reached in case of disagreement. Given the limited text material and the method used, we did not undertake a formal “blinded” assessment of responses. We used MAXQDA 11 and Microsoft Excel 2016 for analysis.

## Results

The online survey resulted in 32 case descriptions. Two had to be excluded from analysis because information on illness trajectory and final diagnosis were not provided. Two more records obviously referred to the same case and were treated as one. This left 29 records with sufficient information for analysis.

### Demographics of participants

Nineteen male and 10 female (34%) doctors provided the reports on the included cases. Four were aged 31–40 years, nine were aged 41–50; nine were aged 51–60, and seven were aged 60 and over. Most participants were general practitioners (20) or GPs in training (2), two were internists, and five did not indicate their specialty. Fourteen participants were recruited via mailing lists connected to the authors’ institutions, three received the link at a local meeting, and 17 gave no indication how they came across the invitation to participate.

### Reported cases

The reported cases involved 15 male and 14 female (48%) patients. Information on age was not provided in four and only vaguely reported in one case. Patient age in the remaining cases ranged from 1.5 to 80 years. The time that had elapsed since the event was less than 1 month in one, 1 to 6 months in four, 7–12 months in five, and 1 to 5 years in nineteen cases.

### Discrepancies between diagnoses

Final diagnoses were more serious than originally assumed in 26 of 29 cases (see Table 2). At first, GPs generally suspected musculoskeletal diseases, uncomplicated infections or deterioration in known conditions. Final diagnoses involved the central nervous system, cardiac diseases, vascular diseases, abdominal diseases and cancer.

**Table 2:** Diagnoses

Severity		Original Diagnosis	Final Diagnosis
Less serious	1	Deep vein thrombosis	Unspecific oedema
Equally serious	2	Bone metastasis from prostate CA (cancer)	Deep vein thrombosis (DVT)
	3	Heart failure	Allergic alveolitis
More serious	4	Unspecific symptoms due to polymyalgia therapy	Decompensated diabetes due to steroids
	5	Constipation	Ileus, patient died
	6	Arthritis	DVT with pulmonary embolism
	7	Muscle strain	DVT with pulmonary embolism
	8	Cervical spine pain	Cerebral venous sinus thrombosis
	9	Cervical spine pain	Unclear, died shortly after visit
	10	Musculoskeletal pain	Bone and liver metastasis in lung cancer
	11	Thoracic spine pain	Perforated peptic ulcer
	12	Chest wall syndrome	Non-ST elevation myocardial infarction
	13	Low back pain	Hypernephroma with renal failure
	14	Non-specific groin pain	Inguinal hernia
	15	Non-specific pain at multiple sites	Pulmonary embolism
	16	Tension headache	Subarachnoid haemorrhage
	17	Migraine with aura	Ischemic stroke
	18	Non-specific vertigo/dizziness	Cerebral lymphoma
	19	Non-specific headache	Subarachnoid haemorrhage
	20	Febrile infection	Meningitis
	21	Diabetic foot ulcer with necrosis	Malignant melanoma
	22	Acute bronchitis	Pulmonary oedema due to mitral insufficiency
	23	Bronchitis	Pulmonary embolism
	24	Cough due to ACE (angiotensin-converting-enzyme) inhibitor	Lung cancer
	25	Chest infection in patient with lung cancer	Pleural effusion, sudden death
	26	Superficial wound with localised skin necrosis	Massive necrosis requiring operation
	27	Hypertensive crisis	Cerebellar infarction
	28	Heart failure or angina pectoris	Leukaemia
	29	Stable angina	Myocardial infarction with subsequent cardiac arrest

In two cases, the final and original diagnoses were of comparable severity. What appeared to be bone metastasis in prostate cancer was in fact deep vein thrombosis, and what seemed to be heart failure was actually allergic alveolitis.

In one case, a patient with a known melanoma and a recently swollen leg had non-specific oedema and not deep vein thrombosis, so the final diagnosis was less severe than the originally suspected disease.

### Clinical trajectory

Most of the patients initially presented with unspecific, benign symptoms. In about one third of cases, a catastrophic event or dramatic deterioration occurred, e.g. abdominal pain suddenly worsened due to a perforated peptic ulcer, or dyspnea at rest resulted from a pulmonary embolism. In three of these cases, patients died shortly afterwards. In more than half the cases, participants did not report permanent damage to the patient.

### Preventability of harm

In retrospect, when a diagnosis is judged to have been wrong, it is usually difficult to establish a causal link between the clinical decision on the one hand and the (negative) outcome for the patient on the other. Given the limited amount of information obtained via the survey, this was especially difficult in the cases discussed here. In order to understand the determinants of regret, we nevertheless attempted to identify whether harm to patients might have been prevented e.g. by reasonable diagnostic measures.

In 10 of the 29 cases, we considered that a correct diagnosis could realistically have been made earlier. This would probably have shortened the patient’s suffering, e.g. when a cerebral lymphoma caused intracranial pressure, or in a case of subarachnoid bleeding. In a few of these 10 cases, a different diagnostic decision might even have changed the fate of the patient. For example, regular blood glucose tests may have prevented a stroke that most likely occurred due to decompensated diabetes under steroids.

In two thirds of cases (19 of 29), a definite diagnosis could not reasonably have been made earlier, as in the case of an elderly woman with assumed constipation that later died of ileus, but who did not follow the physician’s advice to seek admission to hospital.

### Descriptions of regret

In 27 of 29 cases, respondents used strong words to describe an extremely unpleasant experience: “Shock, sorrow”, “I was horrified and concerned.” “I was angry with myself - ashamed at not being able to take on responsibility in front of the patient,” “Powerful feelings of self-reproach (…) feelings of failure.” They gave the impression that they were severely affected by the reported events: “Emotional - disconsolate. ”, “I said I was sorry but was appalled”, “Expressed my dissatisfaction and regret.” According to the definition of regret as “negative emotions connected to cognitions about how past actions might have achieved better outcomes” [1] we summarised these emotions under the term “retrospective regret”. Although most of the cases dated back more than 12 months, the events were vividly remembered and described.

Some factors modified the physicians’ emotions. Participants expressed stronger regret when harm resulting from the reported episode was serious, i.e. permanent disability or even death. However, the preventability of the harm did not appear to be associated with the described dismay.

The reactions of patients, their families and other health professionals also seemed to influence the experienced regret. One GP mentioned disparaging remarks made by hospital doctors, who diagnosed meningitis caused by a *Haemophilus influenzae* infection in an 18-month old vaccinated child. The child had initially presented with signs of a benign respiratory infection. The negative reactions of the hospital doctors had made the situation more difficult to bear for the GP. Moreover, the parents had taken the physician to court and the health insurance company had attempted to recover the costs of treatment. In another case, the GP concerned had tried in vain to contact the patient by phone, which had left the physician with a guilty conscience. Several patients had changed their GP after the event. In another case, rather than blame the doctor, the family had blamed the patient for having refused to go to hospital for several hours despite the GP’s recommendation. Nevertheless, the GP felt distressed by the course of events because he had initially suspected that a perforated peptic ulcer was thoracic spine pain.

The feeling of regret appeared to be somewhat lessened when other physicians were involved in the initial assessment. For example, a 78-year-old female patient was assumed to have non-specific vertigo/dizziness requiring no specific therapy. She consulted not only the reporting physician but also an otorhinolaryngologist and a neurologist, both of whom saw no need for immediate action. However, when her family took her to hospital several weeks later, a CT scan showed an intracerebral lymphoma. Although the reporting physician was “shocked” on learning the outcome, he was relieved not to have been the only doctor to have misdiagnosed the patient.

GPs did not express regret or any other emotional reaction on only two occasions. One of these was the only case that the final diagnosis was less serious than the GP had initially expected. In the other case, the physician concluded that her behaviour had been appropriate, given the nonspecific initial presentation.

## Discussion

### Summary

In our online survey, primary care physicians reported 29 cases in which the final differed from the initial diagnosis. In almost all cases (28 of 29), the final diagnosis was as, or more serious, than the initial one. The course of events, harm to patients and reactions of patients and relatives differed widely. Based on the limited available information, harm to patients was only realistically preventable in about one third of cases. Nevertheless, the vast majority (27 of 29) of physicians expressed deep feelings of regret.

### Comparison with existing literature

In line with other studies, our results show that diagnostic errors have a severe impact not only on patients, but also on physicians. Emotional (and legal) consequences for the physician can be so devastating that the term ‘second victim’ has been coined in the literature [22–24]. Other studies, as well as our analysis, show that the intensity of emotional distress and self-blame is linked to the level of deterioration in patient outcomes [25–27]. The reaction of patients and relatives also influenced our participants’ feeling of regret, as suggested in the literature [25, 28]. However, no studies have investigated the relationship between regret and the preventability of harm.

In this explorative study, our participants articulated regret, including strong emotions such as guilt and shame, irrespective of whether a different clinical course could have prevented harm. This finding is to some degree surprising, but probably shows that physicians strive for and expect perfection of themselves, which is unrealistic in an uncertain clinical environment. Their regret may also be associated with compassion for the patient when, however unavoidably, the correct diagnosis is late and the nature of the illness severe. Whatever the reason for their feelings, physicians experiencing regret draw conclusions that may influence the way they treat future patients. The consequences of experienced regret following diagnostic errors varies. In the case of anticipated regret, several studies have examined the influence it has on the quality of clinicians’ decisions. This is described in a recent review [29]. Anticipated regret may benefit patients by improving the quality of medical decisions and preventing harm, or it may simply make no difference to the quality of decisions [30]. However, reducing the risk of possible regret, as is the case in defensive medicine, may result in additional risks to patients [3]. When shared with colleagues, such experiences may have wider implications for local norms and standards among health care professionals. This issue was also raised in a study on the determinants of defensive medical practices. The study showed that access to an incident reporting system had had a significant impact on most of the defensive medicine measures. Physicians with access to the system, and thereby to their colleagues’ incident reports, practised medicine that was more defensive [31].

### Future support for physicians

The strong feelings of regret following (serious) diagnostic errors are an indication that physicians may benefit from support and the opportunity to talk about what had occurred with colleagues or a supervisor. This could be beneficial after cases where “true” diagnostic errors caused regret, but also where no harm to the patient occurred. Difficult cases and clinical trajectories could also be discussed in peer groups of physicians. This may serve to reassure young physicians in particular, for whom the wide spectrum of cases and a possible fear of excessive demands may be daunting and influence their career planning [32]. Such opportunities may even raise the attraction of working in ambulatory care.

### Strengths and limitations

One strength of our study is that to our knowledge it is the first study to explore regret following diagnostic decisions in primary care. We succeeded in recruiting GPs in early and late stages of their professional lives, and covered a wide variety of experiences.

This study also has limitations. One important limitation is our response rate of less than 1% and that we recruited a convenience sample from the milieu of academic departments of general practice involved in (diagnostic) error research. This limits the generalizability of our findings. Participants may have had contact with the topic beforehand and thus have been more interested in it than the average GP. Our assessment whether harm was preventable or not was necessarily based on sparse data. Even with more information, a posteriori judgements on this issue are difficult and fraught with bias [33, 34]. It also has to be taken into account, that we explicitly asked for the emotional reaction instead of a general reaction to the situation, thus maybe leading to more emotional descriptions. We chose an anonymous online survey with a low participation threshold to gain a first insight into this sensitive topic. Other research methods, e.g. qualitative interviews, are now needed to develop our findings. In such interviews, physicians could be asked for the exact character of and reasons for their regret, e.g. whether their errors had reduced their belief in their own clinical competence, whether they felt guilty about the preventable harm that had occurred following a diagnostic error, and to what extent the regret depended on the feedback of patients. It may also be helpful to investigate what physicians believe could have prevented the diagnostic error, e.g. greater experience, “preparation” for atypical manifestations of diseases through more intensive literature study, having more time per patient, or the availability of further / technical diagnostic measures in their practice.

## Conclusion

High uncertainty and high stakes are characteristic of decisions in medicine [35]. Our results indicate that even when harm was unavoidable, regret can be devastating for clinicians. Despite the relevance of the phenomenon, however, it has received only little attention in scientific and other professional literature. One possible explanation for this blind spot is that physicians like to see themselves as dispassionate implementers of scientific findings. This (self-) image offers no room for feelings of regret. As a result, physicians, as well as their colleagues, patients, and the public at large, often have difficulty accepting that diagnostic assessments always involve some degree of uncertainty. Another explanation is that although regret is widespread and relevant, it is not discussed openly. We would therefore suggest that GPs need more support in dealing with regret than is currently provided. As described in our survey, we need analytical procedures to reflect on cases resulting in undesirable outcomes [19, 36]. It is particularly important to be able to distinguish between ‘true’ diagnostic errors, when harm could have been prevented, and other forms of error. Moreover, GPs need peer support to deal with the emotional impact of their decisions. These measures are especially important for GPs, and might influence their behaviour in future practice.

## Supplementary information

**Additional file 1.** Questionnaire.

## Data Availability

The data that support the findings of this study are not publicly available because they contain information that could compromise research participant privacy. Under certain circumstances, the data may be available from the corresponding author on reasonable request. No additional permission needs to be obtained before the data can be obtained.

## Abbreviations

GP: General practitioner
CA: Cancer
DVT: Deep vein thrombosis
ACE: Angiotensin-converting-enzyme
