# Supplementary material for: Regret among primary care physicians: a survey of diagnostic decisions
Source: BMC Fam Pract. 2020 Mar 17;21:53. doi: 10.1186/s12875-020-01125-w (PMC7079478; doi:10.1186/s12875-020-01125-w)
Supplement: Supplementary file 1 — Additional file 1. Questionnaire. [file 12875_2020_1125_MOESM1_ESM.docx]

# **Additional file 1: Questionnaire**

This questionnaire contains questions about the specific case of a patient that you have experienced. The case should involve a patient or situation in which you made a diagnosis but it later became apparent that another problem (illness) existed than the one you had originally supposed. There may have been underdiagnosis, overdiagnosis or an inappropriate diagnosis, the consequence of which was, at the very least, that treatment was delayed. Perhaps the patient was even harmed.

If you can report on more than one such situation, please choose the most recent example.

The following situations are not of interest in this survey (exclusion criteria):

- Your diagnosis was suboptimal but had no negative consequences for the patient (the latter defined as delayed treatment at the very least).
- The event occurred more than 5 years ago.

| Nr. | Question |
| --- | --- |
| 1 | Please describe your original impression of the patient. At this point, please only describe the situation during the first consultation, as you will be asked to describe the further course of events down below. Please consider:   - Where it took place? Practice, hospital etc. - The patient’s age and gender - Previous diseases and other relevant factors - Current symptoms and findings - Your diagnostic assessment - Any other measures you may have initiated (further examination, referral, hospitalisation, therapy). |
| 2 | How long ago did the contact with the patient occur? |
| 3 | Further course of events: please describe what happened later. |
| 4 | Your reaction. What did you do then? How did you react to the situation emotionally? |
| 5 | Please describe the reaction of the patient and/or relatives. Please think of the following possibilities, which are not mutually exclusive:   - Conversation with the patient and/or relatives - Patient changed doctor - Patient got in contact with a hearing officer/mediator - Civil or criminal proceedings |
| 6 | As a result of the case, is there any way in which you decided to alter your behavior in later decision-making situations? If yes, how? |
| 7 | We have developed an aid to help reflect upon such situations in a structured manner. It involves asking oneself the question whether a rule exists with the help of which it would have been possible to prevent the event occurring. Such rules should have three characteristics. They should be plausible, specific and feasible.  *Plausible:* The rule should take current medical knowledge into consideration. For example, the standard use of Troponin tests for breast pain in the family practice would not be plausible because in the early stages of myocardial infarction, it is not sensitive enough. Indiscriminate use of this test would often wrongly result in patients with an urgent need of treatment not being hospitalized. This criterion ensures that professional knowledge is part of the assessment.  *Specific:* The rule describes specific behavior. An example of the opposite would be resolutions such as “be more careful next time”, “try harder” and such like.  *Feasible:* The regional health system is in a position to cope with the general implementation of the rule. If all patients presenting with breast pain in primary care were hospitalized (emergency admissions, cardiac monitoring units), cardiovascular health care would collapse, as available capacity would not only be utilized by seriously ill patients. Thus adherence to the rule should not lead to large-scale misuse. This criterion also helps promote reflection on whether the implementation of the rule would endanger large groups of patients.  Can you formulate a rule that is plausible, specific and feasible? |
| 8 | What would the rule be? |
| 9 | A new, possibly different assessment of the case: We are interested in knowing whether – having completed this survey – you now think and feel differently about the case. If so, please describe how.  Please consider the professional assessment of the case, your emotional reaction, as well as the consequences (how will I behave in the future?). |
| 10 | Gender, Age  Stage in specialist training  Are you a specialist or planning to become one? In what field?  How many years ago did you complete your specialist training?  Where do you currently work?  How did you find out about our survey? |
